# A Semi-Supervised Attention-Temporal Ensembling Method for Ground Penetrating Radar Target Recognition

**DOI:** 10.3390/s25103138

**Published:** 2025-05-15

**Authors:** Li Liu, Dajiang Yu, Xiping Zhang, Hang Xu, Jingxia Li, Lijun Zhou, Bingjie Wang

**Affiliations:** 1 Key Laboratory of Advanced Transducers & Intelligent Control System, Ministry of Education, College of Physics & Optoelectronics, Taiyuan University of Technology, Taiyuan 030024, China; liuli01@tyut.edu.cn (L.L.); yudajiang1173@link.tyut.edu.cn (D.Y.); zhangxiping1460@link.tyut.edu.cn (X.Z.); xuhang@tyut.edu.cn (H.X.); wangbingjie@tyut.edu.cn (B.W.); 2 Shanxi Intelligent Transportation Institute Co., Ltd., Taiyuan 030024, China; zhoulj-519@163.com

**Keywords:** ground penetrating radar, underground target recognition, deep learning, semi-supervised learning, temporal ensembling, triplet attention

## Abstract

Ground penetrating radar (GPR) is an effective and efficient nondestructive testing technology for subsurface investigations. Deep learning-based methods have been successfully used in automatic underground target recognition. However, these methods are mostly based on supervised learning, requiring large amounts of labeled training data to guarantee high accuracy and generalization ability, which is a challenge in GPR fields due to time-consuming and labor-intensive data annotation work. To alleviate the demand for abundant labeled data, a semi-supervised deep learning method named attention–temporal ensembling (Attention-TE) is proposed for underground target recognition using GPR B-scan images. This method integrates a semi-supervised temporal ensembling architecture with a triplet attention module to enhance the classification performance. Experimental results of laboratory and field data demonstrate that the proposed method can automatically recognize underground targets with an average accuracy of above 90% using less than 30% of labeled data in the training dataset. Ablation experimental results verify the efficiency of the triplet attention module. Moreover, comparative experimental results validate that the proposed Attention-TE algorithm outperforms the supervised method based on transfer learning and four semi-supervised state-of-the-art methods.

## 1. Introduction

With the rapid process of urbanization, urban underground targets are becoming more and more complex, including a large number of underground utilities (e.g., pipes, cables, and rebars). In recent years, road collapse caused by subsurface voids has presented a high incidence trend in major cities all around the world. In addition, unknown buried water, sewage, or gas pipe burst incidents occur sometimes during construction. As a result, accurate recognition and detection of underground targets are of great significance for ensuring the safety of underground spaces in urban areas.

Ground penetrating radar (GPR) is a promising nondestructive testing (NDT) method for detecting underground targets due to its high efficiency, safe operation, good penetrability, and high imaging resolution characteristics. It emits electromagnetic waves and records the reflected signals from material interfaces in the subsurface to infer the target information [1]. GPR has been successfully used in civil engineering fields, such as tunnel lining inspection [2], pavement distress detection [3,4], urban underground pipe detection [5], and so on. However, its data interpretation is still challenging. It primarily relies on the manual work of professional technicians, which is time-consuming and labor-intensive. For example, it may take weeks to interpret GPR data recorded by a vehicle-mounted GPR in just one day. In addition, human judgment is inherently subjective and uncertain. Therefore, it is urgent to develop an automatic GPR target recognition algorithm.

There are mainly two types of automatic target recognition methods: machine learning (ML)-based and deep learning (DL)-based methods. ML-based methods generally extract features from GPR signals and apply machine learning classifiers (e.g., support vector machine (SVM) [4,5,6] and K-nearest neighbors [7]) to recognize underground objects. However, these methods require manual feature extraction according to prior knowledge and expert experience, and the recognition accuracy depends heavily on the quality of features, leading to easy failures in complex field data.

DL-based methods have drawn considerable attention due to their excellent ability to extract high-dimensional features automatically [8]. The most widely used DL model is the convolutional neural network (CNN). Xu et al. [9] used an adaptive one-dimensional CNN and raw GPR signals for hidden distress classification in concrete pavements. Besaw et al. [10] applied the CNN to extract meaningful features from B-scans for detecting buried explosive hazards. Tong et al. [11] presented a cascaded CNN and a multistage CNN for subgrade defect recognition. Ahmed et al. [12] analyzed the classification performance of different deep residual network (ResNet) architectures and DenseNet architectures for underground rebar detection. Rosso et al. [13] explored the vision transformer (ViT) model to classify internal defects of tunnel lining based on B-scan images and presented a preprocessing method based on bidimensional Fourier transform to enhance the classification performance. Three-dimensional (3D) CNNs were also introduced into GPR object classification based on 3D GPR data to recognize targets with similar B-scan reflections [14,15].

Although the aforementioned DL-based methods bring successful results, a significant prerequisite remains that a large amount of labeled data is required for fully supervised learning. However, in GPR fields, especially for defect detection, it is impractical to obtain enough labeled GPR data. First, the defects are intrinsically scarce compared to normal samples. Second, manually labeling GPR data is time-consuming, laborious, and expertise-dependent; even drilling verification is needed.

To solve the problem of insufficient labeled data, researchers usually adopt transfer learning to pretrain a DL model on a public natural image dataset and then fine-tune the model on a small amount of GPR data [16]. However, since GPR data have different features compared to natural images, fine-tuning may not enhance the model’s performance when labeled GPR data are insufficient. Consequently, some researchers transfer the knowledge learned from synthetic data generated by GprMax to the field of GPR data [3]. Moreover, generative adversarial nets (GANs) or variants of GANs have been applied to generate new data to expand the training datasets [2,17]. However, GAN is often hard to converge due to mode oscillation or collapse. In addition, these simulated or generated data still need to be annotated.

Semi-supervised learning (SSL), which employs unlabeled data (data without annotations or predefined labels) and relatively limited labeled data (data with annotations) simultaneously for model training, also has the potential to tackle the issue of inadequate labeled training samples. It has been successfully applied in many DL-based radar applications, such as automatic target recognition using synthetic aperture radars [18,19], radar-based human activity recognition [20,21], radar automatic modulation recognition [22], and ground target recognition using carrier-free ultra-wideband radar [23]. Many semi-supervised DL models have been presented, including GANs [18] and variants of GANs [21], teacher–student models [19], convolutional autoencoder (CAE) [20,23], and pseudo-label [22].

In the field of GPR, G. Reid [24] applied label propagation to detect landmines in a semi-supervised learning manner. However, it is a machine learning method. Todkar et al. [25] presented a semi-supervised modified K-means clustering method to detect small subsurface defects. Liu et al. [26] utilized a semi-supervised deep neural network to realize data inversion for cross-frequency GPR, employing a mean teacher network and a generative adversarial mechanism with random perturbation strategies to improve the robustness of the network. Ma et al. [27] proposed a new prior knowledge-oriented semi-supervised deep learning method for underground pipe detection, which constructs prior structural features to control the quality of pseudo-labeling in the semi-supervised learning process and improve the performance of the model under small samples. Teng et al. [28] integrated a semi-supervised DETR detection model and convolutional augmentation for railway ballast bed defect detection, where the convolutional augmentation module was used to improve the data quality, and the DETR detection model with a confidence filter provided trustworthy pseudo-labels for unlabeled data. However, few studies have focused on semi-supervised deep learning for GPR multi-class target recognition.

In this paper, we propose a semi-supervised learning scheme based on temporal ensembling (TE) [29] with a triplet attention mechanism (abbreviated as Attention-TE) for urban underground multi-class target recognition. It can make full use of abundant unlabeled data to improve classification accuracy and alleviate the burden of annotating a large number of GPR samples. The contribution of this paper is that we first introduce the semi-supervised TE model into GPR target recognition and modify it by incorporating a triplet attention mechanism that can learn spatial and channel information of B-scans more effectively.

The rest of the paper is organized as follows. Section 2 introduces the proposed GPR target recognition based on the semi-supervised Attention-TE. Experimental results from the laboratory and field data are presented in Section 3 and Section 4, respectively. Section 5 provides discussions and the conclusions drawn from the results.

## 2. Underground Target Recognition Based on Semi-Supervised Attention-TE

To solve the problem of limited labeled data, we propose a novel semi-supervised method based on temporal ensembling with the triplet attention mechanism for GPR target recognition. The principle and architecture of temporal ensembling and the triplet attention mechanism are described in detail in this section.

### 2.1. Temporal Ensembling

Temporal ensembling [29] is a kind of self-ensembling semi-supervised learning method. It utilizes multiple versions (ensembles) of a model during training to enhance performance, robustness, and generalization without requiring much labeled data. It has been shown to achieve good results in many areas, such as fake news article detection [30], human activity recognition [31], and medical imaging object detection [32].

**Figure 1:**
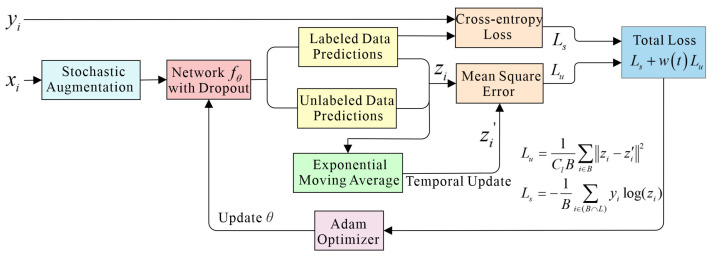
The structure of the temporal ensembling method. The training input (labeled or unlabeled) *x_i_* is fed into a network after the augmentation. The network outputs predictions *z_i_* for all samples. The real label *y_i_* and labeled data prediction are used to calculate supervised cross-entropy loss *L_s_*, whereas both labeled and unlabeled data predictions *z_i_* are combined with previous ensemble predictions *z_i_*’ to calculate unsupervised mean squared error loss *L_u_*. The *z_i_*′ are accumulated by the exponentially moving average method. The total loss is the weighted sum of *L_s_* and *L_u_* and is optimized using the Adam optimizer, and then, the network parameters are updated.

The structure of the temporal ensembling method is shown in Figure 1, and the corresponding training procedure is described in Algorithm 1. Assume that the GPR training dataset {*x_i_*} includes *N* inputs, out of which *M* are labeled. Let *L* consist of the indices of the labeled data, with its element number being *M*. For each *i* ∈ *L*, there is a known correct label *y_i_* ∈ {0, …, *C_l_*−1} where *C_l_* is the number of categories. *B* is the set of minibatch indices. During training, the TE model outputs a prediction vector *z_i_* = *f_θ_*(*g*(*x_i_*)) for each input *x_i_* where *g*(·) is a stochastic augmentation operation and *f_θ_*(·) represents the stochastic neural network with trainable parameters *θ*. The loss function consists of a supervised learning loss, *L_s_,* and an unsupervised learning loss, *L_u_*. For labeled data, the standard cross-entropy is calculated to determine the difference between the real labels, *y_i_,* and predicted ones, *z_i_*. For all training data, the mean square error is used to evaluate the difference between the current predicted outputs *z_i_* and the previous ensemble outputs *z_i_*′. Then the total loss function of the TE model in each minibatch is as follows:

(1) $$\begin{array}{cc} Loss & =L_{s}+w\left(t\right)L_{u}\\ & =-\frac{1}{B}\sum_{i\in (B\cap L)}y_{i}\log(z_{i})+w\left(t\right)\frac{1}{C_{l}B}{\sum_{i\in B}\left\|z_{i}-{z^{\prime }}_{i}\right\|}^{2} \end{array}$$

where *w*(*t*) is a time-dependent weighting function of the epoch *t*. It starts from zero on the first training epoch and slowly ramps up along a Gaussian curve. The total loss is optimized using the Adam optimizer. The supervised loss *L_s_* dominated the total loss in the early training epochs, while unlabeled data contributed more in the later phases.

At the end of each training epoch, the current predictions **z** (a matrix form corresponding to all *z_i_*) are accumulated, and the temporal ensemble outputs (previous predictions) **Z***_t_* and the training targets **z**′ at each epoch *t* are updated by an exponentially moving average (EMA) mechanism:

(2) $$\left\{\begin{array}{l} Z_{t}=\alpha Z_{t-1}+(1-\alpha )z\\ z^{\prime }=\frac{Z_{t}}{1-\alpha^{t}} \end{array}\right.$$

where *α* is a momentum factor between 0 and 1 that controls how far the ensemble reaches into the training history. **Z***_t_* and **z**′ can be initialized as a zero matrix. Through EMA, **Z** contains a weighted average of ensemble outputs from previous training epochs, with recent epochs having higher weights than distant epochs. It can improve the accuracy of pseudo-labels and further enhance the model’s performance.

In our proposed method, the stochastic augmentation *g*(*x*) is performed by adding Gaussian noise. The architecture of the neural network will be described in Section 2.3. The momentum factor *α* is taken as 0.6.
 **Algorithm 1:** Temporal ensembling pseudocode **Input:** *x_i_*: Training sample, *i*∈*N*    *y_i_*: Labels for labeled samples, *i*∈*L*    *L*: Set of training sample indices with known labels     *f_θ_*(·): Neural network with trainable parameters *θ*
     *g*(*·*): Stochastic augmentation function
     *w*(*t*): Weighting function
     *α*:  Momentum factor, 0 < *α* < 1 Initialization:  $Z\leftarrow 0_{\left[N\times C_{l}\right]}$                        ▷Initialize ensemble predictions              $z^{\prime }\leftarrow 0_{\left[N\times C_{l}\right]}$                       ▷Initialize target vectors
**1**. **For** *t* in [1, epochs] **do**
**2**.    **For** each minibatch *B*
**do**
**3**.        *z_i_*
← *f_θ_* (*g*(*x_i_*))                ▷Evaluate network outputs
**4**.        $Loss\leftarrow -\frac{1}{B}\sum_{i\in (B\cap L)}y_{i}\log(z_{i})$           ▷Supervised loss component                            $+w\left(t\right)\frac{1}{C_{l}B}{\sum_{i\in B}\left\|z_{i}-{z^{\prime }}_{i}\right\|}^{2}$     ▷Unsupervised loss component
**5**.        update *θ* via Adam optimizer     ▷Update network parameters
**6**.    **end for**
**7**.    Z←αZ+(1−α)z                      ▷Accumulate ensemble predictions
**8**.    $z^{\prime }\leftarrow Z/\left(1-\alpha^{t}\right)$                         ▷Construct target vectors by bias correction
**9**. **   end for**
**10**. **return** *θ*

### 2.2. Triplet Attention

To improve the feature extraction performance, we introduce a recently presented triplet attention mechanism [33] into the TE model. Unlike the squeeze-and-excitation (SE) attention module, which only pays attention to channel information, triplet attention can effectively capture spatial and channel information with an almost parameter-free attention mechanism. It accounts for cross-dimensional interactions between the channel dimension and spatial dimensions and, thus, can capture rich, discriminative feature representations of the input to improve classification performance.

**Figure 2:**
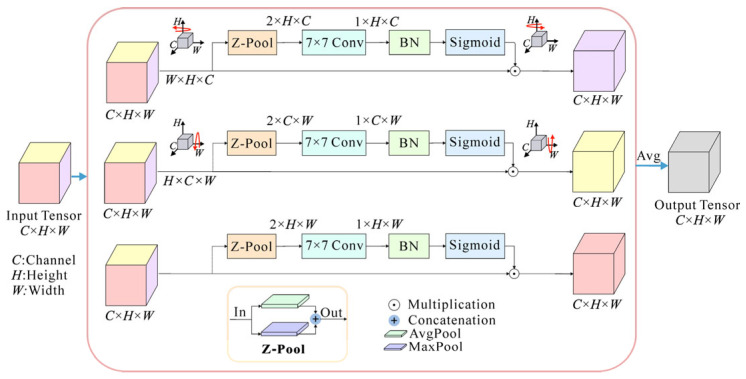
The structure of the triplet attention module. The input tensor enters three parallel branches. The first branch is used for computing attention weights across the channel dimension *C* and the spatial height dimension *H*. The second branch is responsible for channel dimension *C* and spatial width dimension *W*. The third branch is used to build spatial attention (*H* and *W*). Finally, the weights are aggregated by simple averaging. Z-Pool: concatenation of the max-pooling (MaxPool) and the average-pooling (AvgPool); Conv: convolutional layer; BN: batch normalization.

The structure of triplet attention is shown in Figure 2. It utilizes a parallel three-branch structure where one branch is used to build spatial attention and two branches are responsible for capturing cross-dimensional interactions between the channel dimension and spatial dimensions.

In the input tensor *χ* ∈ ℝ*^C^*^×*H*×*W*^, *C*, *H*, and *W* are the dimensions of channel, height, and width, respectively. In the first branch, the triplet attention module establishes the interactions between the height dimension and the channel dimension. The input *χ* is rotated 90° counterclockwise along the *H* axis to obtain a rotated tensor *χ*_1_ with the size of *W* × *H* × *C*. Then, *χ*_1_ passes through a Z-pool layer and reduces to a 2 × *H* × *C* tensor. After that, this tensor is squeezed into an intermediate tensor of size 1 × *H* × *C* through a standard convolutional layer (Conv) with a 7 × 7 kernel size and a batch normalization (BN) layer. Next, this intermediate tensor passes through a sigmoid activation layer to generate the final attention weights. The attention weights are subsequently applied to *χ*_1_, which is then rotated 90° clockwise along the *H* axis to obtain *χ_HC_* with the same size as the original input *χ*. The process of this branch can be represented by:

(3) $$\chi_{HC}=R_{H}^{+}\left(\chi_{1}\sigma \left(\mathrm{ConvBN}\left(Z-\mathrm{pool}\left(\chi_{1}\right)\right)\right)\right),\;\chi_{1}=R_{H}^{-}\left(\chi \right)$$

where $R_{H}^{+}$ and $R_{H}^{-}$ denote clockwise and counterclockwise rotation along the *H* axis, respectively; *σ* is the sigmoid activation function; ConBN represents the combination operation of convolution and batch normalization; and Z-pool is a compound pooling operation by concatenating the results of the max-pooling (MaxPool) and the average-pooling (AvgPool) operation, which is responsible for reducing the first dimension of the tensor *χ*_1_ from *W* to 2.

Similarly, in the second branch, *χ* is rotated counterclockwise by 90° along the *W* axis to get the rotated tensor *χ*_2_. Then *χ*_2_ passes through the attention branch to capture the dependency between the (*C*, *W*) dimensions. The output of this branch is denoted as *χ_CW_*. For the last branch, the input tensor *χ* is directly input into the branch without rotation to capture the spatial information from the (*H*, *W*) dimension of the input tensor. The resulting tensor is defined as *χ_HW_*. These calculation processes can be described by the following equations:

(4) $$\begin{array}{l} \chi_{CW}=R_{W}^{+}\left(\chi_{2}\sigma \left(\mathrm{ConvBN}\left(Z-\mathrm{pool}\left(\chi_{2}\right)\right)\right)\right),\;\chi_{2}=R_{W}^{-}\left(\chi \right)\\ \chi_{HW}=\chi \sigma \left(\mathrm{ConvBN}\left(Z-\mathrm{pool}\left(\chi \right)\right)\right) \end{array}$$

where $R_{W}^{+}$ and $R_{W}^{-}$ denote clockwise and counterclockwise rotation along the *W* axis, respectively. To realize the fusion of spatial attention and channel attention information, the output tensor *χ*′ of the triplet attention module is obtained by simply averaging the refined tensors generated by three branches:

(5) $$\chi^{\prime }=\frac{1}{3}\left(\chi_{HC}+\chi_{CW}+\chi_{HW}\right)$$

With this design, the triple attention module can effectively capture the interactions between spatial and channel dimensions in the input tensor, thus improving the model’s ability to understand features.

### 2.3. Attention-TE Method

**Figure 3:**
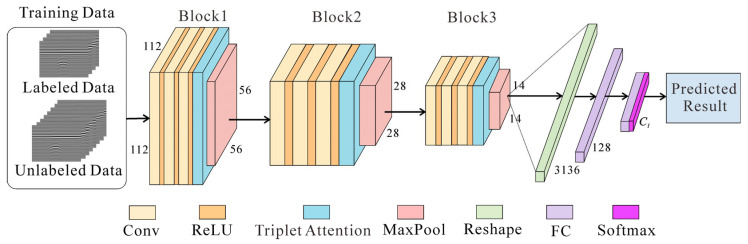
The architecture of the network. A small amount of labeled data and a large amount of unlabeled data are used to train the model. The label is a number in the range of [0, *C_l_* − 1]. Three blocks are used to extract features. FC layers are responsible for integrating features extracted by three blocks and then making decisions that are passed through a Softmax function to generate class predictions.

The framework of the Attention-TE method is shown in Figure 3, and the corresponding parameters are listed in Table 1. This network is the embodiment of the neural network *f_θ_*(·) in Figure 1. In the training process, limited labeled data and sufficient unlabeled data are used together to train the model. Labeled data are data that have been assigned a label or category. Here, the label is represented by a number in the range of 0 to *C_l_* − 1. Different numbers correspond to different categories of the targets. For example, there are three categories to be classified into, such as cable, void, and pipe, and we define the label 0 for cable, 1 for void, and 2 for pipe. Unlabeled data, on the other hand, are data that do not have any labels or categories assigned to them.

The Attention-TE model consists of three convolutional blocks for feature extraction, two fully connected (FC) layers for feature mapping, and a Softmax output layer. Each convolutional block has three convolutional (Conv) layers, followed by a ReLU activation layer, a triplet attention module, and a max pooling layer (MaxPool). The input size is 1 × 112 × 112. After passing through three blocks, the size of the input is changed to 32 × 56 × 56, 64 × 28 × 28, and 16 × 14 × 14, respectively. These three blocks are responsible for extracting hierarchical features, firstly focusing on the edges and textures in the whole image (e.g., the ground clutter, the background clutter, and the target reflection curve), then gradually concentrating on the reflection curve from the target (e.g., hyperbola). Next, the extracted local feature matrix is reshaped into a 1 × 3136 vector and imported into two consecutive FC layers with 128 units and *C_l_* units to integrate into global representations. Finally, the Softmax layer outputs a predicted result. This result is then used to compute the loss function to update the network parameters. After the model is trained well, it can provide classification results for the testing data.

## 3. Laboratory Experiments

### 3.1. Laboratory Data Acquisition

**Figure 4:**
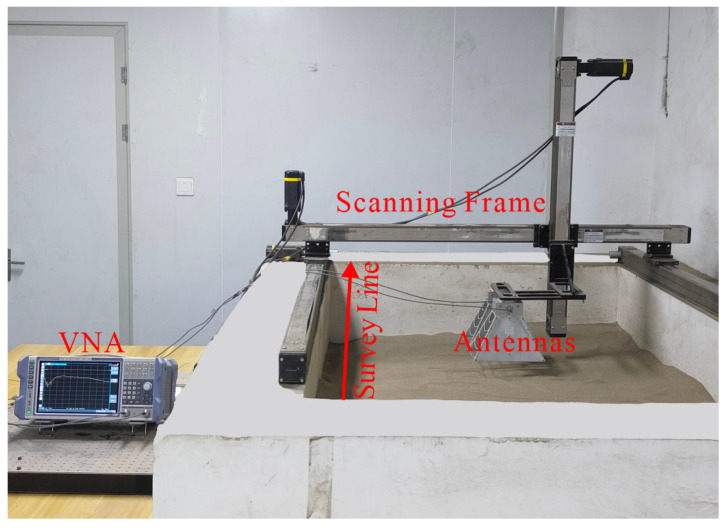
The experimental scene.

The laboratory data were collected by our self-established GPR system in a sandbox, as shown in Figure 4. A sandbox with dimensions of 2.0 × 1.2 × 0.8 m was filled with compacted dry sand. The relative dielectric permittivity of the dry sand is about 3, and its conductivity is negligibly small in the GPR frequency range. We use the sandbox to physically simulate simple, realistic scenarios. Although these simple scenarios differ from actual detection environments, the collected data have similar geometric features. A vector network analyzer (VNA) generated a stepped-frequency continuous-wave signal with a 1.8–5 GHz frequency range and a step size of 8 MHz. A customized mechanical scanning frame was installed on the sandbox and can be implemented in a three-dimensional automatic motion using a microcontroller and a stepping motor. A pair of wideband horn antennas with an 18 cm interval was linked with the scanning frame and moved along the survey line with a step size of 2 cm. In the experiment, a target (i.e., a metal pipe, a plastic pipe, or an acrylic box) was randomly buried in the sand at different depths and positions. The acrylic box was used to simulate the void. The background scenarios without targets were also collected. The antennas were placed about 5–10 cm above the dry sand surface. For each B-scan, 72 traces were recorded, with 400 sample points per trace. Finally, a total of 1080 B-scans were obtained, including 310 for metal pipes, 330 for plastic pipes, 200 for acrylic boxes, and 240 for the background. For each B-scan, we saved it as a gray image with 496 × 369 pixels.

**Figure 5:**
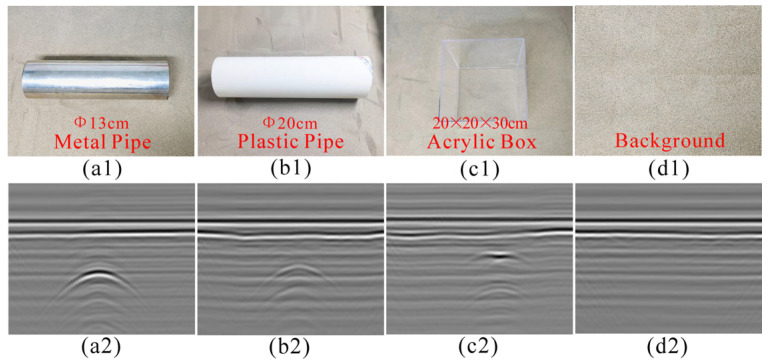
Targets and corresponding B-scan images in the experiments: (**a**) metal pipe; (**b**) plastic pipe; (**c**) acrylic box; (**d**) background (without targets).

Figure 5 shows the targets used in the experiments and the corresponding B-scan images. It can be found that strong ground clutter was distinct in all images, and the four B-scan images have different visual features. The reflections from the pipes have typical hyperbolic features, but the plastic pipe exhibits weaker intensity than the metal pipe. The reflection from the acrylic box’s upper surface exhibits a roughly flat top with half-hyperbola curves on both sides due to a flat interface reflection from the upper surface and two overlapping diffracted waves from the box edges, while the reflection from the lower surface presents a weak hyperbola.

All the images of each category were randomly divided into the training set (60%, 648 samples), the validation set (20%, 216 samples), and the testing set (20%, 216 samples). For each image, we selected a target region to obtain a 336 × 336 square image. Since 648 samples are not enough to train the model, we augmented the training set to 2592 samples by shifting operations. Specifically, in the images with 496 × 369 pixels, we shifted the target region selection box right by 50 pixels and down by 30 pixels, respectively, and combined them for right-down shifting. Then, three extra sets of 648 images were obtained, which were integrated with the original training set to construct an expanded training set. Table 2 lists the number of datasets after data augmentation. All images were resized to 112 × 112 to match the size of the input unit of the network. Note that the images were directly sent into the network without any preprocessing.

### 3.2. Network Training for Laboratory Data

The proposed Attention-TE network was performed on a cloud platform deployed with deep learning environments, with an NVIDIA GeForce RTX 4090 24 GB GPU and AMD EPYC 7452 CPU. Pytorch 1.12.1 was used for deployment. The Adam optimizer with a learning rate of 1 × 10^−4^ was utilized for training all architectures from scratch. The batch size was 32, and the epoch was 300.

**Figure 6:**
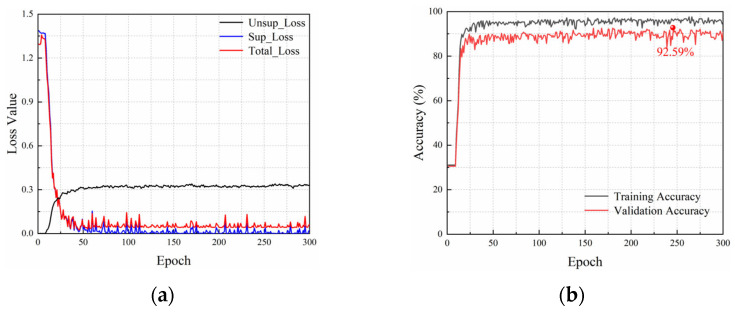
Loss curves and accuracy curves under a 10% labeling rate: (**a**) loss curves; (**b**) accuracy curves.

We define the labeling rate as the ratio of the labeled data to the training data. Figure 6 shows the loss curves and accuracy curves of the proposed Attention-TE method under a 10% labeling rate. The loss function of the network consists of supervised loss (Sup_Loss) and unsupervised loss (Unsup_Loss), so there are three curves in Figure 6a. With the increase in training epochs, the unsupervised loss curve grows fast and then becomes stable, whereas the supervised loss curve drops rapidly and then vibrates within a narrow range. Therefore, the total loss value decreases sharply in the first 50 epochs and then fluctuates to some extent. After about 125 epochs, the curve exhibits a stable trend, and the loss has converged. As shown in Figure 6b, the accuracy on the validation set rises rapidly to above 80% in the first 25 epochs and then increases slowly within 125 epochs. After that, it tends to stabilize but fluctuates slightly around 90%. The highest accuracy of the Attention-TE reaches 92.59%.

### 3.3. Classification Results of Laboratory Data

**Figure 7:**
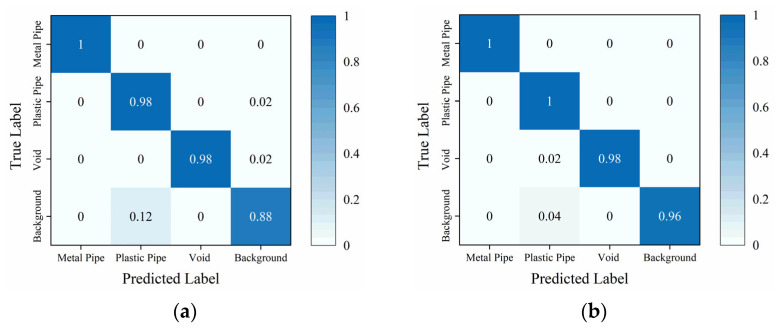
The confusion matrix under a 20% labeling rate: (**a**) TE; (**b**) Attention-TE.

Figure 7 illustrates the confusion matrix of the traditional TE and the proposed Attention-TE. When just 20% of the training data are labeled, two methods can classify metal pipe, plastic pipe, and void with high accuracy (above 98%). The traditional TE misjudges the background images as plastic pipe images with low accuracy because when the reflection of the plastic pipe is too weak, the background images and plastic pipe images have similar features. However, the Attention-TE presents a significant improvement in identifying the background, reaching 96% from 88% of the TE due to the strong feature extraction ability of the triplet attention. It misjudges 2% of the void images as plastic pipe images because when the void’s reflection is weak, the flattened apex feature may not be distinct, leading to a hyperbolic feature similar to the plastic pipe’s reflection. The average accuracy of the TE for the four categories is 96%, whereas the Attention-TE reaches a 98.5% accuracy.

**Figure 8:**
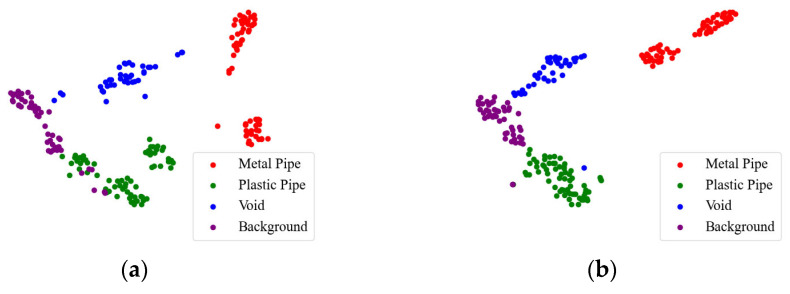
Visualization of recognition results by t-SNE: (**a**) TE; (**b**) Attention-TE.

To intuitively show the effect of the triplet attention mechanism, we use t-distributed stochastic neighbor embedding (t-SNE) [34] to visualize the recognition results under a 20% labeling rate. As shown in Figure 8, the GPR images are clustered into four categories by the red, green, blue, and purple points. The red (metal pipe) dots and blue (void) dots in the TE result are scattered, and the green (plastic pipe) dots are intertwined with the purple (background) dots. In the Attention-TE result, each category has a relatively compact region. Only one purple dot and one blue dot are present in the green area, which means a background image and a void image are misjudged as plastic pipes. The proposed Attention-TE has a better clustering result than the traditional TE model, corresponding to the higher accuracy, as shown in Figure 7.

Further, Table 3 gives the recognition accuracy of four categories under different labeling rates. To remove possible biases, we repeated all the experiments ten times to obtain the average accuracy and standard deviation. Obviously, the accuracy per category and the average accuracy of the four categories show an upward trend as the labeling rate increases, but the increment gradually decreases. For example, when the labeling rate grows from 5% to 20%, the proposed method achieves a 6.96–4.29–2.91% improvement in average accuracy, whereas when the labeling rate is over 20%, the accuracy only increases slightly, probably because the network has learned sufficient information from 20% labeled data. As for each category, metal pipe images have the highest accuracy due to strong reflections, whereas the background images have the worst recognition results since they are incorrectly predicted to be plastic pipes or voids.

### 3.4. Ablation Experiments for Laboratory Data

To verify the effectiveness of the triplet attention, we performed ablation experiments on the traditional TE, the TE with an SE module (TE + SE), and our proposed Attention-TE. All the experiments were also repeated ten times. Table 4 lists the classification results of three models under different labeling rates and the growth compared to the baseline (TE), where the highest values in average accuracy are in bold and the growth values are presented after the arrow symbols. The classification performance of all models improves as the labeling rate increases. Moreover, the attention module added to the TE network can benefit the average accuracy, and the triplet attention module can achieve higher improvement over the SE module. For example, when the labeling rate is 20%, the average accuracy of the Attention-TE improves from 96.28% of the TE to 98.37%, an improvement of 2.09%, whereas the TE with an SE module has a similar average accuracy as the TE, only a 0.23% growth.

### 3.5. Comparison with State-of-the-Art Methods for Laboratory Data

To demonstrate the superiority of the proposed Attention-TE with the limited labeled data, we compare it with four state-of-the-art SSL methods, including a pseudo-label [35], semi-supervised convolutional autoencoder (SCAE) [20], semi-supervised GAN (SGAN) [36], and Ladder network [37]. In the pseudo-label method, we first use it to generate pseudo-labels for the unlabeled data, then retrain the VGG16 model with the labeled and pseudo-labeled training data. As for the semi-supervised CAE, we employ a three-layer encoder and decoder. In the SGAN, the discriminator has three convolutional layers, and the generator has five convolutional layers for optimizing the results. Besides that, a supervised method based on transfer learning is also implemented. The VGG16 model is first pretrained on the ImageNet dataset and then fine-tuned on the GPR labeled dataset.

To evaluate the classification performance quantitatively, we use four quantitative indicators, including accuracy, precision, recall, and F1-score. Table 5 gives the comparison results under a 10% labeling rate. It can be found that the Ladder network presents the worst performance. The pseudo-label method has better results than SCAE and SGAN. The proposed method outperforms other methods in terms of all four evaluation indicators. It achieves an average accuracy of 91.17%, higher than 89.45% of the supervised method using the VGG16 model. F1-score is the harmonic mean of precision and recall and can more comprehensively reflect the classification performance of the model. As shown in Table 5, the proposed method also has the highest F1-score, about 92.20%, indicating that the Attention-TE model has better performance in identifying underground targets.

## 4. Field Experiment

### 4.1. Field Data Acquisition

**Figure 9:**
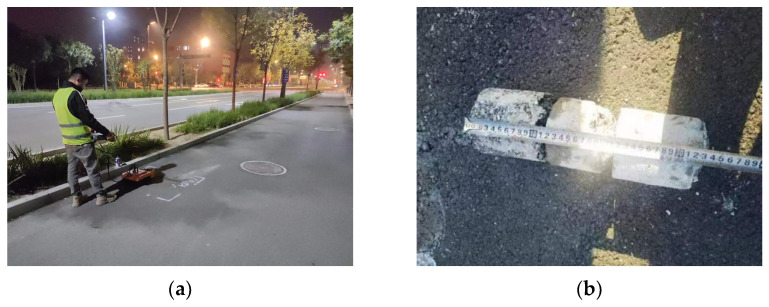
(**a**) Photos of field data collection; (**b**) an example of a core sample.

**Figure 10:**
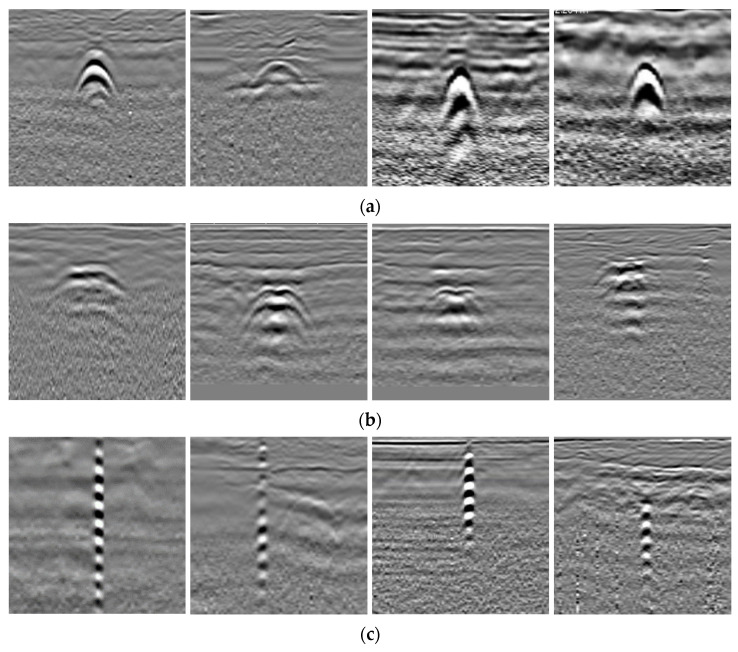
GPR field data examples: (**a**) pipe; (**b**) void; (**c**) cable.

The field data were collected using an LTD-2600 GPR from pavements in Beijing, China. Figure 9a shows the field data collection. The targets in the field data include pipes, voids, and cables. All the data were distinguished and labeled by experts and preprocessed using the IDSP7 software by time-zero correction, direct wave removal, and automatic gain control. The data corresponding to the voids were verified by the core sampling method, as shown in Figure 9b. The B-scan images, including one target, were cropped from the preprocessed GPR data. In total, there were 2940 B-scans, including 1000 for pipes, 940 for voids, and 1000 for cables. These B-scans were resized to 112 × 112 and divided into the training set, validation set, and testing set in a ratio of 8:1:1. In the training set, some images were randomly selected for annotation according to the labeling rate. Table 6 lists the number of datasets, and Figure 10 gives some examples of the field data. It can be observed that the field data are more complex and diverse. The data of the same category have different patterns due to various collection environments and the target’s geometry and dimensions. The reflection curves of pipes are regular hyperbolas, and the cables present several stronger reflection hyperbolas in the vertical direction. The voids exhibit irregular hyperbola-like curves but with a flatter top and multiple distinct reflections.

### 4.2. Network Training for Field Data

**Figure 11:**
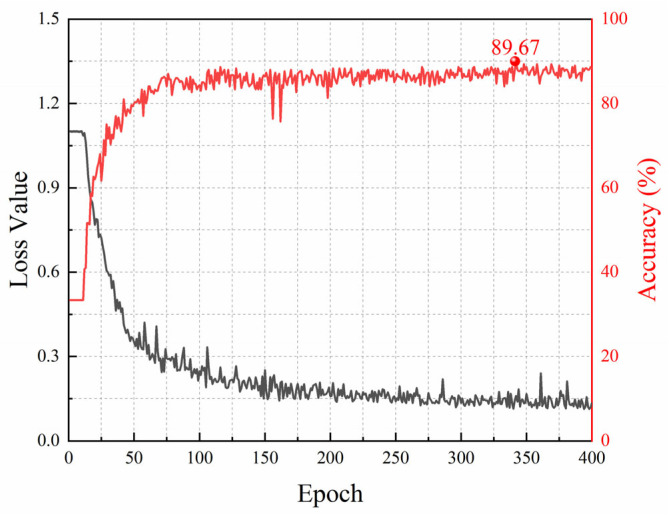
The loss curve and accuracy curve under a 20% labeling rate for field data.

We trained the model using the field dataset. The training hyperparameters employed were the same as those in the laboratory experiments, except that the epoch was set to 400. Figure 11 shows the training loss curve and the accuracy curve on the validation set when the labeling rate is 20%. The training loss drops sharply in the first 100 epochs and levels off gradually. After about 300 epochs, the loss has converged. The accuracy on the validation set increases rapidly to above 80% in the first 100 epochs and then grows slowly within 200 epochs. After that, it tends to stabilize but fluctuates slightly around 88%. The highest accuracy reaches 89.67%.

### 4.3. Classification Results of Field Data

**Figure 12:**
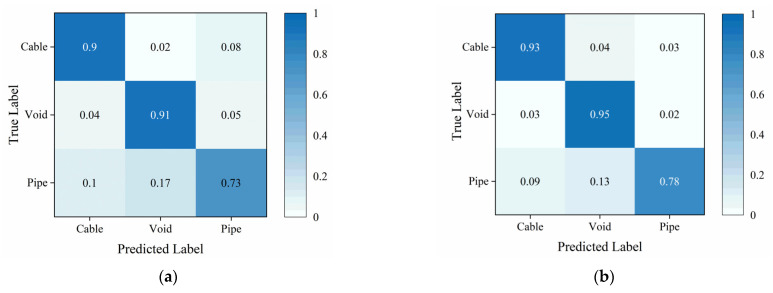
The confusion matrix under a 20% labeling rate for field data: (**a**) TE; (**b**) Attention-TE.

**Figure 13:**
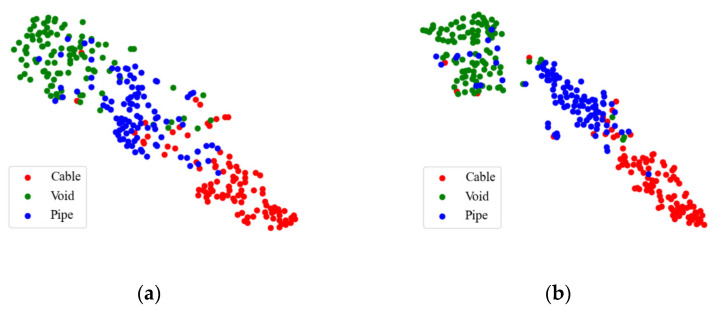
T-SNE results under a 20% labeling rate for field data: (**a**) TE; (**b**) Attention-TE.

Figure 12 depicts the confusion matrix of the traditional TE and the proposed Attention-TE under a 20% labeling rate for field data. We can find that the two methods can classify cables and voids with high accuracy (above 90%) and misjudge the pipes as voids and cables with low accuracy. However, the Attention-TE presents an improvement in identifying three categories compared with the TE. The average accuracy of the TE for the three categories is 84.67%, whereas the Attention-TE reaches 88.67% accuracy. We also use t-SNE to visualize the results. As shown in Figure 13, the GPR images are clustered into three categories by the red, green, and blue points. The blue (pipe) dots in the TE result are separated and intertwined with the red (cable) and green (void) dots. In the Attention-TE result, each category has a compact region. Some blue dots are present in the green and red areas; that means pipes are misjudged as voids or cables, corresponding to confusion matrix results, because cylinder-shaped voids have similar B-scan reflections as pipes and cables. Despite this, the proposed Attention-TE has a better clustering result than the traditional TE model.

### 4.4. Ablation Experiments for Field Data

**Figure 14:**
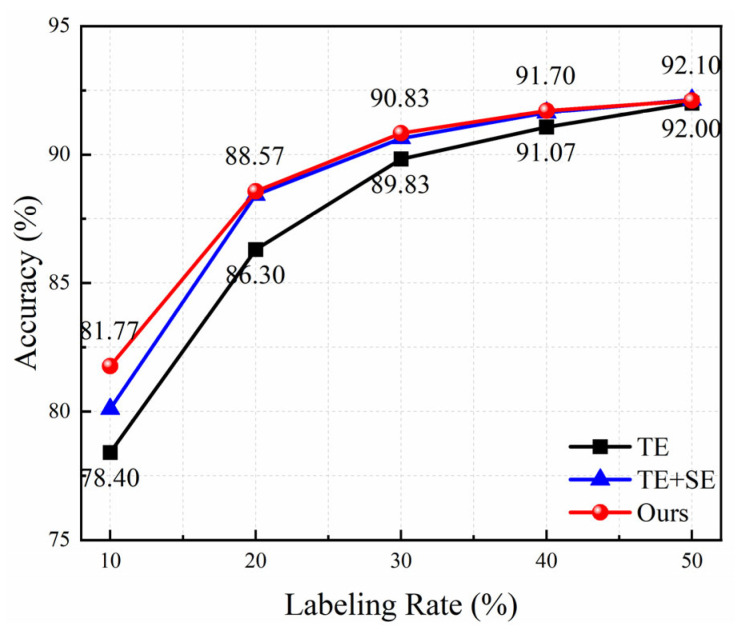
Ablation experimental results under different labeling rates for field data.

Figure 14 presents the average accuracy of three models with the increase of the labeling rate for field data. Obviously, the accuracy of all methods increases rapidly at first and then slowly. The attention module is conducive to the improvement in recognition performance. The proposed method has the highest accuracy value under different labeling rates, demonstrating that the triplet attention mechanism can capture richer information to enhance the model’s capability. Especially when the labeling rate is 10%, the average accuracy increases from 78.4% to 81.77%, a nearly 3.4% improvement; when the labeling rate is 20%, it increases from 86.3% to 88.57%. The increment becomes smaller as the labeling rate grows. The accuracy achieves above 90% under the labeling rate of 30%, which can satisfy the requirements of practical applications.

### 4.5. Comparison with State-of-the-Art Methods for Field Data

Table 7 depicts the comparison results of different semi-supervised methods under a 20% labeling rate. Similar to the results of laboratory data, the proposed method has the best recognition performance, followed by the supervised method using the VGG16 model and the semi-supervised pseudo-label method. The average accuracy reaches 88.57%, which is lower than that of the laboratory experimental result due to complex environments, different types of pavement, and various shapes of targets. In addition, the precision, recall, and F1-score of the proposed method present the highest values compared to other methods. All these results demonstrate that making full use of the GPR unlabeled data can, indeed, help learn the intrinsic distribution and structure of the data, thus improving recognition performance with limited labeled data.

## 5. Conclusions and Discussions

To address the problem of insufficient labeled training data, we propose a GPR target recognition method based on semi-supervised temporal ensembling, which can fully utilize a large amount of unlabeled GPR data and limited labeled data. We also modify the traditional TE model by introducing the triplet attention mechanism. The triplet attention has the ability to capture inter-spatial and channel information to enrich extracted features and, thus, improve classification performance. Experimental results on laboratory data and field data have demonstrated that the proposed Attention-TE method can identify underground objects with above 90% accuracy under a less than 30% labeling rate. Especially when the labeling rate is 10%, it reaches an average accuracy of 91.17% for laboratory data. Ablation experimental results have proven the efficiency of the triplet attention module, which achieves higher accuracy compared with the traditional TE under different labeling rates. Moreover, compared with the supervised method based on transfer learning and four semi-supervised methods, including pseudo-label, SCAE, SGAN, and Ladder network, the proposed method has a higher recognition performance. Therefore, it can be applied in GPR automatic underground object classification with limited labeled data, thus promoting the applicability of deep learning in actual road subsurface inspection.

Note that our proposed method has a simple structure and no requirement for model pretraining or class balance, which is conducive to practical applications. The most potential limitation of the proposed method is that when the data volume is large, it will take a very long time to train the model since it requires recording previous prediction results to update the new prediction result according to the EMA mechanism, as shown in Equation (2). Fortunately, the data volume in GPR fields is relatively small compared with that in computer vision. In addition to that, the proposed method was validated using controlled laboratory and field data. It is recommended to further provide independent expert validation or large-scale real-world validation to fully assess its generalization and robustness. However, it is difficult to carry out. This limitation is typical in semi-supervised learning for GPR, where collecting extensive ground-truth data is particularly challenging.

Through the comparison results in Table 4 and Figure 14, it is not difficult to see that the average accuracy of laboratory data is significantly higher than that of field data due to different detection environments. For laboratory data, collection conditions are consistent and simple (only one target with regular geometry is buried in the dry sand), while the field data are collected from different urban pavements. Since the actual materials in the subsurface are diverse and heterogeneous, and the targets in the same category are various, the reflection curves of the same target have different properties according to geometric configuration and dimensions, as shown in Figure 10. Therefore, the inherent complexity and diversity of field data result in lower accuracy. Nevertheless, our proposed method outperforms other methods for field data.

The current study has only examined the most common types of targets (e.g., voids, pipes, and cables) in the subsurface of urban pavement. In the future, we will consider other types of underground targets and collect more diverse data from the field to further validate the proposed algorithm. We will also discuss model optimization or other improvement strategies to further enhance the classification performance and the generalization ability of the model. In addition, the impact of different preprocessing methods on the recognition results in complex environments should also be considered.

## Figures and Tables

**Table 1:** Parameters of the proposed Attention-TE.

Block1	Block2	Block3	FC
Conv 3 × 3, F = 32, S = 1 Conv 3 × 3, F = 32, S = 1 Conv 3 × 3, F = 32, S = 1 Triplet Attention MaxPool 3 × 3, F = 32, S = 2, D = 0.5	Conv 3 × 3, F = 64, S = 1 Conv 3 × 3, F = 64, S = 1 Conv 3 × 3, F = 64, S = 1 Triplet Attention MaxPool 3 × 3, F = 64, S = 2, D = 0.5	Conv 3 × 3, F = 32, S = 1 Conv 3 × 3, F = 16, S = 1 Conv 3 × 3, F = 16, S = 1 Triplet Attention MaxPool 3 × 3, F = 16, S = 2, D = 0.5	FC1 128 FC2 *C_l_*

F-Filters, S-Stride, D-Dropout.

**Table 2:** The number of B-scan images used in the laboratory experiments.

Target	Total	Training	Validation	Testing
Metal pipe	868	744	62	62
Plastic pipe	924	792	66	66
Void	560	480	40	40
Background	672	576	48	48
Total	3024	2592	216	216

**Table 3:** Average accuracy (%) of the four categories under different labeling rates.

Labeling Rate	Metal Pipe	Plastic Pipe	Void	Background	Average Accuracy
5%	94.35 ± 4.04	89.09 ± 3.56	74.25 ± 5.28	79.17 ± 10.1	84.21 ± 2.91
10%	99.52 ± 0.78	97.27 ± 1.72	88.50 ± 4.89	79.38 ± 3.47	91.17 ± 0.92
15%	100.0 ± 0.00	98.03 ± 1.76	99.00 ± 1.75	84.79 ± 2.95	95.46 ± 0.72
20%	99.36 ± 0.83	99.24 ± 1.07	98.00 ± 1.58	96.87 ± 2.03	98.37 ± 0.70
25%	99.68 ± 0.68	99.24 ± 1.64	99.00 ± 2.42	96.67 ± 1.76	98.65 ± 0.62

**Table 4:** Ablation experimental results in average accuracy (%) under different labeling rates.

Labeling Rate	TE	TE + SE	Ours
5%	81.75 ± 3.20	82.62 ± 2.66↑0.87	**84.21 ± 2.91↑2.46**
10%	90.53 ± 0.55	91.05 ± 1.21↑0.52	**91.17 ± 0.92↑0.64**
15%	94.35 ± 0.46	94.55 ± 0.70↑0.20	**95.46 ± 0.72↑1.11**
20%	96.28 ± 0.56	96.51 ± 0.33↑0.23	**98.37 ± 0.70↑2.09**
25%	97.10 ± 0.69	97.54 ± 0.29↑0.44	**98.65 ± 0.62↑1.55**

**Table 5:** Comparison of experimental results under a 10% labeling rate for laboratory data.

Methods	Pseudo-Label [35]	SCAE [20]	SGAN [36]	Ladder Network [37]	VGG16	Ours
Accuracy (%)	88.11 ± 2.30	82.78 ± 3.11	78.47 ± 3.54	76.63 ± 2.01	89.45 ± 0.29	**91.17 ± 0.92**
Precision (%)	90.28 ± 3.06	83.95 ± 3.75	80.47 ± 3.24	77.79 ± 2.22	89.82 ± 0.36	**93.27 ± 0.72**
Recall (%)	88.11 ± 2.30	82.78 ± 3.11	78.47 ± 3.54	76.63 ± 2.01	89.45 ± 0.29	**91.17 ± 0.92**
F1-score (%)	89.18 ± 2.59	83.36 ± 3.42	79.45 ± 3.33	77.21 ± 2.11	89.63 ± 0.32	**92.20 ± 0.76**

**Table 6:** The number of B-scan images used in the field experiments.

Target	Total	Training	Validation	Testing
Pipe	1000	800	100	100
Void	940	740	100	100
Cable	1000	800	100	100
Total	2940	2340	300	300

**Table 7:** Comparison of experimental results under a 20% labeling rate for field data.

Methods	Pseudo-Label	SCAE	SGAN	Ladder Network	VGG16	Ours
Accuracy (%)	83.60 ± 3.77	77.30 ± 1.85	63.70 ± 3.96	65.63 ± 1.67	85.93 ± 0.83	**88.57 ± 1.75**
Precision (%)	84.06 ± 3.55	78.60 ± 1.53	64.08 ± 3.89	66.45 ± 1.63	86.12 ± 0.94	**89.06 ± 1.41**
Recall (%)	83.60 ± 3.77	77.30 ± 1.85	63.70 ± 3.96	65.63 ± 1.67	85.93 ± 0.83	**88.57 ± 1.75**
F1-score (%)	83.83 ± 3.65	77.94 ± 1.58	63.89 ± 3.92	66.04 ± 1.64	86.03 ± 0.88	**88.81 ± 1.57**

## Data Availability

Dataset available on request from the authors.
